# Mitochondria DNA Change and Oxidative Damage in Clinically Stable Patients with Major Depressive Disorder

**DOI:** 10.1371/journal.pone.0125855

**Published:** 2015-05-06

**Authors:** Cheng-Chen Chang, Shaw-Hwa Jou, Ta-Tsung Lin, Te-Jen Lai, Chin-San Liu

**Affiliations:** 1 The Institute of Medicine, Chung Shan Medical University, Taichung, Taiwan; 2 Department of Psychiatry, Changhua Christian Hospital, Changhua, Taiwan; 3 Department of Psychiatry, Taichung Tzuchi Hospital, The Buddhist Tzuchi Medical Foundation, Taichung, Taiwan; 4 Department of Medicine, Buddhist Tzu Chi University, Hualien, Taiwan; 5 Department of Life Sciences, National Chung Hsing University, Taichung, Taiwan; 6 Vascular and Genomic Research Center, Changhua Christian Hospital, Changhua, Taiwan; 7 Graduate Institute of Integrated Medicine, College of Chinese Medicine, China Medical University, Taichung, Taiwan; 8 Department of Psychiatry, Chung Shan Medical University Hospital, Taichung, Taiwan; University of Texas Health Science Center at San Antonio, UNITED STATES

## Abstract

**Background:**

To compare alterations of mitochondria DNA (mtDNA) copy number, single nucleotide polymorphisms (SNPs), and oxidative damage of mtDNA in clinically stable patients with major depressive disorder (MDD).

**Methods:**

Patients met DSM-IV diagnostic criteria for MDD were recruited from the psychiatric outpatient clinic at Changhua Christian Hospital, Taiwan. They were clinically stable and their medications had not changed for at least the preceding two months. Exclusion criteria were substance-induced psychotic disorder, eating disorder, anxiety disorder or illicit substance abuse. Comparison subjects did not have any major psychiatric disorder and they were medically healthy. Peripheral blood leukocytes were analyzed to compare copy number, SNPs and oxidative damage of mtDNA between the two groups.

**Results:**

40 MDD patients and 70 comparison subjects were collected. The median age of the subjects was 42 years and 38 years in MDD and comparison groups, respectively. Leukocyte mtDNA copy number of MDD patients was significantly lower than that of the comparison group (p = 0.037). MDD patients had significantly higher mitochondrial oxidative damage than the comparison group (6.44 vs. 3.90, p<0.001). After generalized linear model adjusted for age, sex, smoking, family history, and psychotropic use, mtDNA copy number was still significantly lower in the MDD group (p<0.001). MtDNA oxidative damage was positively correlated with age (p<0.001) and MDD (p<0.001). Antipsychotic use was negatively associated with mtDNA copy number (p = 0.036).

**Limitations:**

The study is cross-sectional with no longitudinal follow up. The cohort is clinically stable and generalizability of our result to other cohort should be considered.

**Conclusions:**

Our study suggests that oxidative stress and mitochondria may play a role in the pathophysiology of MDD. More large-scale studies are warranted to assess the interplay between oxidative stress, mitochondria dysfunction and MDD.

## Introduction

Mitochondria play an important role in energy metabolism [1,2]. It is well known that mitochondrial oxidative phosphorylation system generates free radicals and the electron transport chain itself is vulnerable to damage by free radicals [3]. Human mitochondrial DNA (mtDNA) is prone to oxidative injury because mtDNA is not protected by histones and mitochondria generate reactive oxygen species (ROS) during ATP synthesis [4]. Mitochondrial dysfunction results from alterations in biochemical cascade and the damage to the electron transport chain has been suggested to be an important factor in the pathogenesis of a range of psychiatric disorders, such as major depressive disorder (MDD) [5]. The exact pathophysiology of major depression is not clearly understood. A Small but growing body of evidence, including animal study, muscle biopsy, and imaging data, supports the role of mitochondrial dysfunction in MDD. Gardner et al. [6] showed a significant decrease of mitochondrial ATP production rates and mitochondrial enzyme ratios in muscle compared to controls in MDD patients. Madrigal et al. [7] reported that mitochondrial respiratory chain was inhibited in rat brains after chronic stress for 21 days. In humans, PET studies of cerebral blood flow or glucose metabolism in patients with MDD have found reduced blood flow and metabolic rate in prefrontal cortex, anterior cingulate gyrus and basal ganglia [8]. Vawter et al. [9] analyzed mtDNA copy number in post-mortem brains in patients with psychiatric disorders, there was a trend towards decreased mtDNA copy number in patients bipolar disorder compared to controls after controlling for the strong effects of agonal duration. However, there is still no conclusive *in vivo* evidence about the relationship between mtDNA variations and oxidative damage in MDD. Kato et al. proposed mitochondrial dysfunction hypothesis involving the 10398A and 5178C genotypes in the pathophysiology of BD [10,11], whether 10398A and 5178C mtDNA polymorphisms play a role in MDD is unknown.

Mitochondrial abnormalities in psychiatric disorders are not exclusively observed in the brain [12–14]. Leukocytes have been shown to be a good cell model for studies of mitochondrial function [13,15]. By analyzing peripheral blood leukocytes, we hypothesized that alteration of mtDNA copy number, single nucleotide polymorphisms (SNPs) and oxidative damage of mtDNA between clinically stable depressed patients and a normal comparison group.

## Methods and Materials

### 2.1. Subjects

Participants (N = 40) who met DSM-IV diagnostic criteria for MDD were recruited from the psychiatric outpatient clinic at Changhua Christian Hospital, Taiwan from January to December in 2009. Diagnosis of MDD was made by chart record and the patient was interviewed by a board certified psychiatrist using Mini-International Neuropsychiatric Interview [16]. These subjects had to be clinically stable (defined by scale of clinical global impression of illness, CGI) and their medications had to be unchanged for at least the preceding two months. The index score for CGI was one or two. Exclusion criteria were substance-induced psychotic disorder, eating disorder, anxiety disorder or illicit substance abuse. After explaining the purpose of this study and obtaining written informed consent, the following data were collected: age, gender, family history of MDD in first-degree relatives, body mass index (BMI), cigarette smoking and current medication use.

The comparison group was composed of 70 persons aged 25–55 years who were included in our previous study of mtDNA copy number in human leukocytes [13]. We recruited these subjects from the health screening clinic at Changhua Christian Hospital and reviewed their chart records. The subjects were interviewed to make sure they had no history of major psychiatric disorders, were non-smokers and were not taking psychotropics. All participants were medically healthy at study entry with no history of chronic medical illness, no indication of acute infection and not pregnant. The study was approved by the Institutional Review Board of Changhua Christian Hospital and was conducted in accordance with the Helsinki Declaration.

### 2.2. Determination of copy number and oxidative damage in human leukocyte mtDNA

Venous blood samples (5mL) were collected from patients and controls. The leukocyte DNA was extracted from peripheral white blood cell by the Gentra Puregene DNA kit (Qiagen, Germany) to avoid platelet contamination. The mtDNA content, which is usually measured by copy number, has been used as an index of stability of mitochondrial genes [17]. Leukocyte mtDNA copy number was determined with quantitative-PCR (Q-PCR) and the procedure was modified by our previous report. [13,18] Briefly, PCR was performed by amplification of the ND1 gene in mtDNA and the β-globin gene in nuclear DNA from sampled DNA, and standard regression analyses were used to derive the mtDNA copy number.

The content of 8-OHdG in mtDNA was determined by a quantitative real-time PCR method and was presented as ΔCt [19]. After treated mtDNA with human 8-Oxoguanine glycosylase (hOGG1), the 8-OHdG residue was removed to form an abasic site. ΔCt value was determined by calculating the difference between cycle threshold (Ct) value before and after treatment with hOGG1.The larger the ΔCt was, the more abundant the 8-OHdG and more oxidative DNA damage the sample had. The value of ΔCt was calculated for each sample as an index of oxidative stress to mtDNA.

### 2.3. The polymorphisms of G10398A and A5178C in human leukocytes mtDNA

Kato et al. found that the rates of G1039A and A5178C genotype were significantly higher in patients with bipolar disorder even when paternally transmitted cases were excluded [10,11], whether such polymorphisms in patients in MDD are unknown. We screened the G10398A and A5178C polymorphisms by PCR and direct sequencing. Two primer sets were used for PCR amplification: (1) mtF10126 (5'- GACTACCACAACTCAACGGC-3') and mtR10629 (5'-GGGAGTGGGTGTT-GAGGG- 3'), (2) mtF4881 (5'- CCCATCTCAATCATATACCA- 3') and mtR5539 (5'-TCTTGGTCTG TATTTAACCTA- 3'). The PCR reaction mixture contained 20 ng DNA, 375 mmol/L of each dNTP, 50 nmol of each primer, 1X PCR buffer, and 2.5U Taq DNA polymerase (BD Biosciences, San Jose, CA) in a final volume of 20 μL. The amplification conditions were 35 cycles of 94°C for 30 seconds, 56°C for 30 seconds, and 72°C for 60 seconds. Then the PCR products were sequenced using an ABI 3130xl genetic analyzer and a BigDye Terminator v1.1 cycle sequencing kit (Applied Biosystems, Foster City, CA).

### 2.4. Determination of 4,977-bp deletion of mtDNA (dmtDNA^4977^)

Many different types of somatic mtDNA mutations have been observed, with the dmtDNA^4977^ being the most common in humans [20]. The dmtDNA^4977^ occurs frequently in tissues of high oxygen demand and low mitotic activity, e.g. brain, heart, or skeletal muscle. The common deletion levels were increased in the certain brain region of patients with bipolar disorder compared to controls, but not in schizophrenia or MDD [21]. The 4,977-bp deletion of mtDNA was detected in human leukocytes by PCR. More details were described in our previous study [22]. Briefly summarizing, using the forward primer 5-GCCCGTATTTACCCTATAGC-3 and the reserve primer 5-GGGGAAGCGAGGTTGACCTG-3), we amplified a 423-bp DNA fragment from mtDNA with 4,977-bp deletion. Each PCR run was done in a 50 μL reaction mixture containing 100 ng of template DNA, 200 μM dNTPs, one unit of FastStart Taq DNA Polymerase (Roche Molecular Biochemicals, Mannheim, Germany), 20 pmol of each primer, 50 mM KCl, 1.5 mM MgCl_2_, and 10 mM Tris-HCl (pH 8.3). A total of 35 cycles of PCR was performed for each sample in a MJ Research PTC-200 Thermo Cycler (GMI, Inc., Minnesota, USA). The first cycle consisted of 3 min denaturation at 95°C, 5 min annealing at 55°C and 1 min primer extension at 72°C. The other PCR conditions were as follows: denaturation at 95°C for 40 sec, annealing at 55°C for 40 sec and extension at 72°C for 50 sec. The PCR products were detected by electrophoresis on a 1.5% agarose gel at 100 V for 40 min and under UV trans-illumination after EtBr staining.

### 2.5. Statistical analysis

Data were analyzed using SPSS17 (SPSS Inc., Chicago, IL, USA). Continuous variables in the two groups showed a non-parametric distribution and they were compared using Mann-Whitney U test. Categorical variables were analyzed using the χ^2^ test or Fisher’s exact test. Generalized linear model was applied to test the relationship between illness and copy number or mtDNA∆Ct after adjusting for age, gender, medical illness, smoking and psychotropic medications. All statistical tests performed were 2-tailed. The significance level was set at 0.05.

## Results

**Fig 1:**
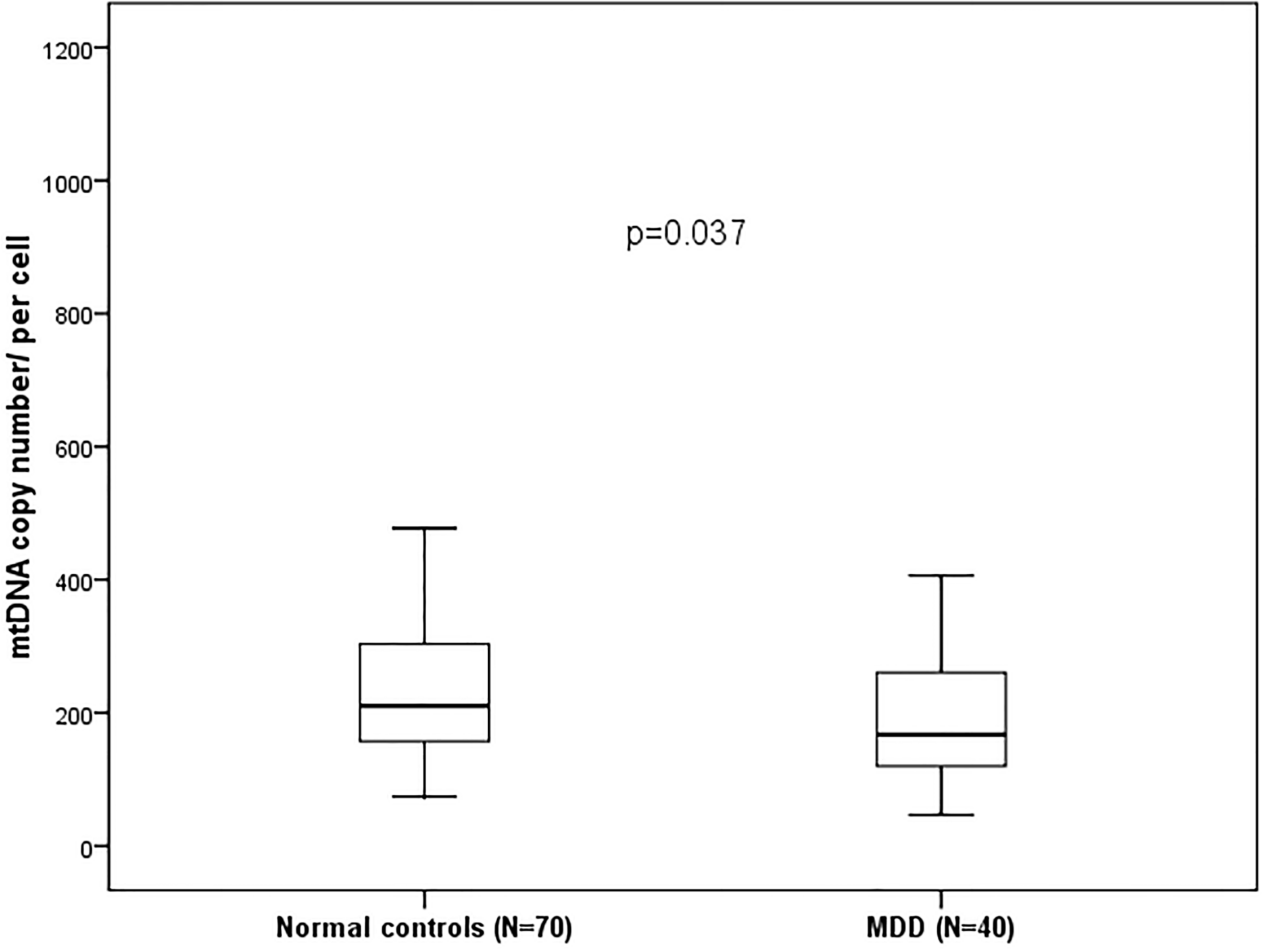
Comparison of mtDNA copy number/per cell between normal controls and MDD patients.

We recruited 40 (36.4%) males and 70 (63.6%) females. Table 1 showed the characteristics of these participants. 28 (40%) males and 42 (60%) females were included in the comparison group, while 12 (30%) males and 28 (70%) females in MDD group (p = 0.29). The median age of the subjects was 42 years and 38 years in MDD and comparison groups, respectively (p = 0.039). 32 (80%) MDD patients received treatments for more than one year. The BMI between the two groups were similar (22.96 vs. 22.08). The prescribed medicines were as follows: 4 were on tricyclic antidepressants (10%), 18 on selective serotonin reuptake inhibitors (45%), 12 on serotonin–norepinephrine reuptake inhibitors (30%), 8 on noradrenergic and specific serotonergic antidepressants (20%), 9 on antipsychotics (22.5%), 33 on benzodiazepines (82.5%). Leukocyte mtDNA copy number of MDD group was significantly lower than that of control group (p = 0.037) (Fig 1). The MDD patients had significantly higher rates of smoking (p = 0.001). No common 4977-bp deletion in mtDNA was detected and the SNPs of mtDNA C5178A and A10398G were similar in both groups.

**Table 1:** Demographic and clinical characteristics between comparison and MDD groups.

	Comparison group (N = 70)	MDD group (N = 40)	P value
Male	28 (40%)	12 (30%)	0.29
Female	42 (60%)	28 (70%)	
Age(median±IQR)	38.00±16.50	42.00±18.75	0.039
Family history illness	0	13 (32.5%)	<0.001
Smoking	0	7 (17.5%)	0.001
BMI	22.96±2.74	22.08±3.33	0.205
TCA	-	4 (10.0%)	-
SSRI	-	18 (45.0%)	-
SNRI	-	12 (30.0%)	-
NaSSA	-	8 (20.0%)	-
Antipsychotics	-	9 (22.5%)	-
BZDs	-	33 (82.5%)	-
B blocker	-	11 (27.5%)	-
Copy number (median±IQR)	214.27±163.65	167.27±142.86	0.037
mtDNA△Ct (median±IQR)	3.90±3.59	6.44±6.03	0.001
mtDNA C5178A(+)	11(15.7%)	8(20%)	0.57
mtDNA A10398G(+)	24(34.3%)	17(42.5%)	0.39

BMI: body mass index.

TCA: tricyclic antidepressant.

SSRI: selective serotonin reuptake inhibitor.

SNRI: selective serotonin-norepinephrine inhibitor.

NaSSA: noradrenergic and specific serotonergic antidepressant.

BZDs: benzodiazepines.

IQR: interquartile range.

**Fig 2:**
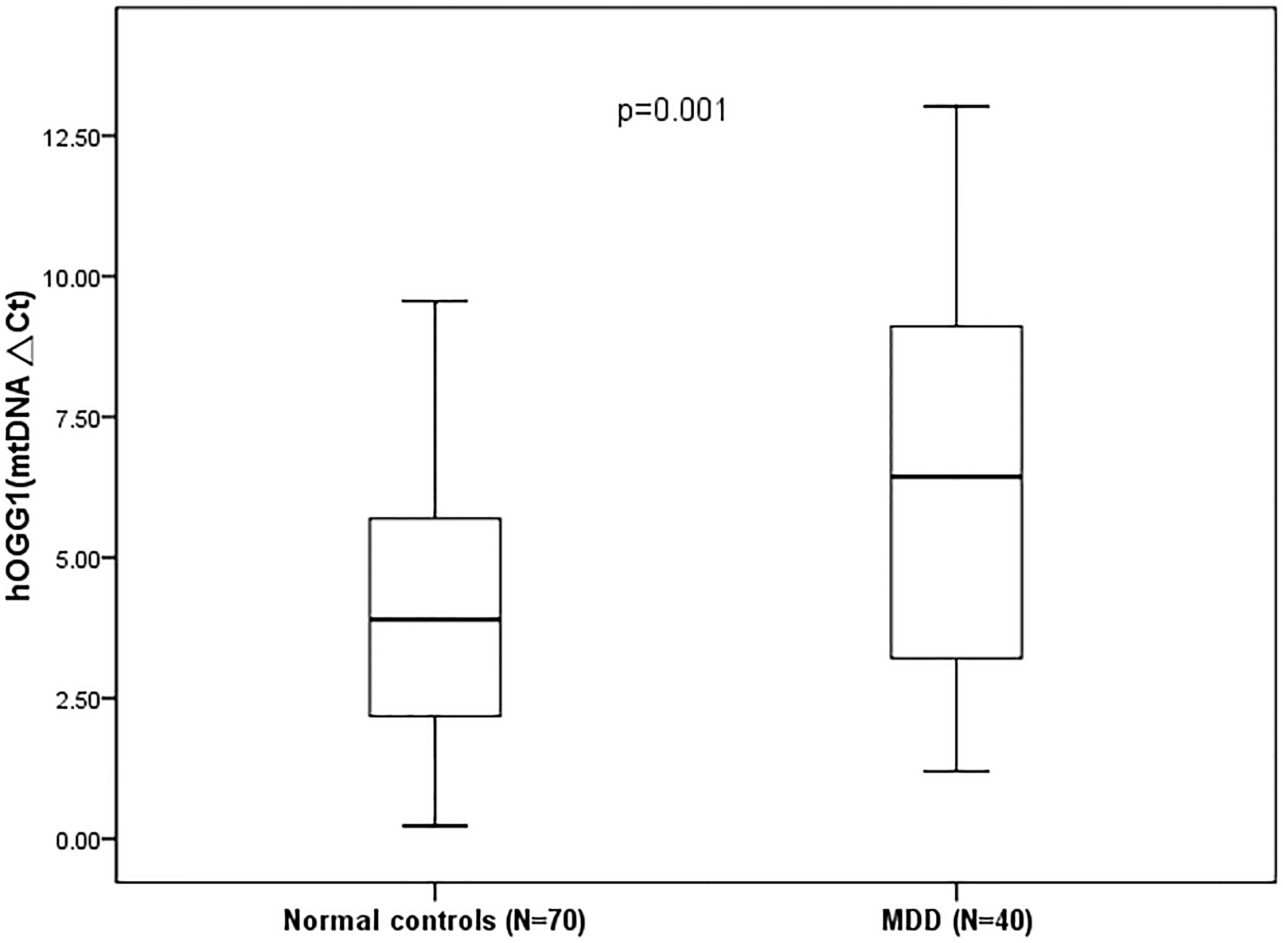
Comparison of mtDNA△Ct between normal controls and MDD patients.

The clinical alteration in 8-OHdG content in mitochondrial DNA was measured as mtDNA∆Ct. Higher mtDNA∆Ct suggested more mitochondrial oxidative damage. MDD patients had significantly higher mitochondrial oxidative stress than the control group (6.44 vs 3.90, p = 0.001) (Fig 2). In a generalized linear model adjusting for age, sex, family history, smoking and medication use, mtDNA copy number of MDD group was significantly lower than that of comparison group (p = 0.001). MtDNA△Ct was significantly increased with MDD group (p<0.001) and age (p<0.001). In terms of medication use, antipsychotics were negatively associated with mtDNA copy number (p = 0.036) (Table 2).

**Table 2:** Generalized linear model to identify independent clinical variables associated with copy number and mtDNA∆Ct.

	mtDNA Copy number			mtDNA ∆Ct		
	B	95%CI	p value	B	95%CI	p value
Age	1.217	-1.019~3.453	0.286	0.110	0.168~0.052	<0.001
Male/female	4.112	-47.311~55.534	0.875	0.017	-1.159~1.192	0.978
MDD/comparison	-190.458	-302.989~-77.928	0.001	7.046	3.060~11.031	0.001
Family history(+)	68.889	-11.829~149.606	0.094	-0.447	-2.465~1.571	0.664
Smoking(+)	30.171	-59.552~119.895	0.510	0.945	-1.579~3.470	0.463
TCA	-48.552	-156.813~59.709	0.379	-2.255	-6.257~1.746	0.269
SSRI	112.093	-4.413~228.599	0.059	-3.429	-6.944~0.086	0.056
SNRI	34.211	-56.282~124.705	0.459	-2.798	-6.069~0.472	0.094
NaSSA	2.462	-84.663~89.587	0.956	-3.411	-6.771~0.065	0.053
Antipsychotics	-86.965	-168.293~-5.637	0.036	-0.786	-3.081~1.510	0.502
BZDs	67.456	-10.442~145.354	0.090	-0.796	-3.294~1.702	0.532
β blockers	28.754	-53.614~111.121	0.494	-0.404	-2.458~1.651	0.700

TCA: tricyclic antidepressant.

SSRI: selective serotonin reuptake inhibitor.

SNRI: selective serotonin-norepinephrine inhibitor.

NaSSA: noradrenergic and specific serotonergic antidepressant.

BZDs: benzodiazepines.

## Discussion

To our knowledge, this was the first in vivo study to detect and compare differences in mtDNA variations and mtDNA△Ct change between a normal population and stable MDD patients in Pubmed. We found that leukocyte mtDNA copy number of MDD group was significantly lower than that of comparison subjects. MtDNA△Ct, as a proxy of oxidative damage, was significantly higher in the MDD group. However, no common mtDNA deletion was detected and the SNPs of mtDNA C5178A and A10398G were similar in both groups.

Abnormal mtDNA number content has been associated with disturbed mitochondrial function and increased oxidative stress [23]. Kim et al. found decreased mtDNA content in leukocytes of elderly patients with depressive symptoms [24]. This result was compatible with ours while our sample mainly comprised of adults with MDD. Their study evaluated depressive symptoms only, while our study used standard tools for diagnosis, further enhancing the validity of our finding. In contrast with our results, one postmortem study showed a strong association between longer agonal duration and increased mtDNA copy number in the dorsolateral prefrontal cortex [9]. But after controlling for agonal duration, the authors identified no copy number difference between patients with major depressive disorder and controls. Contrary to our finding, He et al. found a negative association between leukocyte mtDNA copy number and MDD in young adults [25]. This may be because their patients were younger than ours, 60% of them were first-episode MDD, and nearly 40% of them unmedicated. Another possible explanation of their findings is the “threshold hypothesis of mtDNA copy number control” [17]. He et al. postulated that as people grew older, their compensation ability weakened, and their threshold downregulated, finally resulting in a decrease in mtDNA copy number.

Oxidative stress and mitochondrial injury have been postulated as mechanisms in the pathogenesis of MDD [26,27]. Mitochondrial oxidative phosporylation system generates free radicals and the electron transport chain itself is vulnerable to oxidative damage by free radicals. Oxidative damage to proteins or mtDNA will change protein-protein interaction and induce epigenetic modification [3]. The mtDNA copy number in leukocyte is decreased by oxidative stress [13]. This was consistent with our findings that in MDD patients oxidative damage was significantly increased and mtDNA copy number was decreased.

Many different types of somatic mtDNA mutations have been observed, with dmtDNA^4977^ being the most common in humans [28]. However the dmtDNA^4977^ mutation cannot yet be detected in much lower amounts in fast replicating cells such as blood leukocytes [29]. This may explain why no dmtDNA^4977^ was detected in our samples. In regard to SNP changes, several groups have investigated SNP in the mitochondrial genome and bipolar disorder and major depressive disorder. The mtDNA 5178 A>C variant of the complex I subunit 1 (MT-ND1) gene was analyzed in leukocytes and the 5178C genotype was significantly more common in bipolar disorder compared to controls in cases with a maternal family history [11]. Two studies of mtDNA sequence variants and mutations in post-mortem tissue in MDD have not identified any significant findings [30,31]. In our study mtDNA C5178A and A10398G polymorphisms showed no difference between MDD and comparison groups. Ethnic difference might contribute to our findings. It is also possible that MDD is much more heterogeneous and the contribution of gene-environmental interactions cannot be overlooked.

In our study, mtDNA oxidative damage still increased in MDD group even after controlling. It may be that depression contributes to oxidative damage through increase in the production of ROS. Another possibility is that depression does not increase production or exposure to ROS but rather decreases repair of damaged mtDNA. After controlling related variables, age was still positively correlated with increased oxidative damage of mtDNA. This was compatible with mitochondrial free radical theory of aging [32]. Oxidative damage caused by reactive oxygen species would contribute to the impaired physiological function and reduced life-span. Oxidative DNA damage, primarily that affecting mtDNA, increases in the aged brain [33,34]. Liu et al. [13] found that in healthy subjects, the mtDNA copy number in the leukocyte seemed to correlate positively with age before an individual reaches middle age, and progressively shifted to negative correlation thereafter. Possibly because our subjects were around middle age, leukocyte mtDNA copy number was significantly lower in our patients than in normal controls.

Psychotropic medications may influence mitochondrial function in varying degrees.

Research suggests antipsychotic treatment may be related to increased antiapoptotic effects mediated by mitochondrial signals [35]. B cell leukaemia/ lymphoma 2, which encodes an antiapoptotic neuroprotective protein on the mitochondrial outer membrane, is commonly induced by lithium and valproate in one animal study [36]. After controlling with related factors, our study showed antipsychotic use significantly decreased mtDNA copy number. It seems antipsychotic drugs may compromise the expression of mitochondria. However we did not record dose and duration of drug use, further experiments are needed to clarify the relationship between psychotropic drugs and mitochondrial changes *in vivo*.

This study has several limitations. First, the cross-sectional study design does not give any inference about causal relationship between mtDNA variations, oxidative damage and MDD. Second, many psychotropic medications may interfere with mitochondrial function. The impacts of psychotropic medications on mitochondrial function are mainly from animal studies and are still inconclusive [35,36]. Third, using CGI to define stability rather than a standard tool (such as the Hamilton Depression Rating Scale) is a limitation of the study. Our patients were relatively stable and stayed on the same drug dose for at least two months, this reflected clinical daily practice. Fourth, the amount of mtDNA quantified by only one region of mtDNA (ND1) also limits the generalization of our results to other mtDNA region. Using human leukocytes to measure mtDNA function is convenient and noninvasive.

The exact mechanisms involved in MDD are still unknown. Further studies with larger samples may provide additional data regarding the possible involvement of oxidative damage and mitochondria in the pathophysiology of MDD. It is important that psychiatrists retain a high level of suspicion for mitochondrial dysfunction in their patients of MDD.
